# Investigation of the Relationship Between Self‐Consciousness and Autobiographic Memory in Individuals with Autism Spectrum Disorders with LiveCam and fNIRS

**DOI:** 10.1002/brb3.70349

**Published:** 2025-02-28

**Authors:** Yesim Unveren, Mevhibe Saricaoglu, Ece Zeynep Karakulak, Lütfü Hanoğlu

**Affiliations:** ^1^ Life With Children Center Istanbul Turkey; ^2^ Research Institute for Health Sciences and Technologies (SABITA), Regenerative and Restorative Medicine Research Center (REMER), Clinical Electrophysiology, Neuroimaging and Neuromodulation Lab Istanbul Medipol University Istanbul Turkey; ^3^ Vocational School, Program of Electroneurophysiology Istanbul Medipol University Istanbul Turkey; ^4^ Department of Neurology Istanbul Medipol University Istanbul Turkey

**Keywords:** autism spectrum disorder, autobiographical memory, fNIRS, LiveCam, self

## Abstract

**Introduction:**

Autism spectrum disorder (ASD) is a prevalent neurodevelopmental condition with implications for self‐consciousness and autobiographical memory. This study investigates the relationship between self‐consciousness, autobiographical memory, and associated neurobiological structures in ASD.

**Methods:**

There were two groups: autism (*N* = 4) and a control group without autism symptoms (*n* = 8), which underwent a 2‐day LiveCam camera recording and subsequent functional near‐infrared spectroscopy (fNIRS) tasks with familiar (“old”) and unfamiliar (“new”) images. Results revealed distinct hemodynamic patterns in brain regions related to self‐awareness, memory recall, and language comprehension.

**Results:**

In the control group, the presentation of “old” images elicited higher oxyhemoglobin concentration changes in the frontopolar, orbitofrontal, dorsolateral prefrontal cortex (DLPFC), and temporal areas. For “new” images, differences extended to DLPFC, frontal and temporal areas, somatosensory cortex, and subcentral areas between groups. Autobiographical memory tests indicated that individuals with ASD struggled to recall specific memories and exhibited differences in memory themes and narrative length compared to the control group. While emotional elements were preserved, sensory details were often overlooked.

**Conclusion:**

The findings suggest that challenges in accessing and integrating autobiographical memories and self‐related information may impact the development of a stable self‐identity in ASD. The study underscores the importance of understanding the neural basis of self‐consciousness and memory in autism, offering insights into potential areas for intervention and support.

## Graphical abstract

The study comprises two groups: autism spectrum disorder (ASD) and control group. The study consists of the following stages: (1) informing and evaluating individuals and providing LiveCam camera training; (2) spending 2 days with LiveCam camera recording; (3a) two types of images were established. The “old” images were chosen from visuals that the person witnessed and recorded by the camera. “New” images were chosen from the images obtained from other participants; (3b) the recording of fNIRS data 36 h after camera registration is complete; (3c) after the fNIRS data were obtained, autobiographical memory tests and theory of mind tests were applied. (4) *Results*: *fNIRS recordings on old and new images*: Significant differences in oxyhemoglobin concentration changes were found between the ASD and control groups when viewing both “old” and “new” images, with the control group showing higher levels in various brain regions. *Comparison of HbO within groups*: No significant differences in cortical activation were observed in the ASD or control groups when comparing responses to “old” versus “new” images. *Autobiographical memory tests*: Significant differences were found between the ASD and control groups, with the ASD group reporting lower levels. *Theory of mind test findings*: Significant differences were identified between the ASD and control groups in their ability to order story sequences and understand false beliefs accurately, indicating difficulties in social cognition for the ASD group. *Correlation of HbO and behavioral data*: In the ASD group, no significant correlations were found between oxyhemoglobin concentration changes and autobiographical memory data, whereas the control group exhibited significant correlations in many channels.

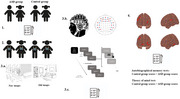

## 1 Introduction

Autism spectrum disorder (ASD), a neurodevelopmental condition, entails sensory sensitivities, social communication challenges, stereotypical behaviors, and limited attention (Y. Li and Yu 2016). “Autism” originates from the Greek word “autos,” meaning “self.” Cognitive neuroscience reveals shifts in self‐related consciousness in ASD individuals. Earlier views portrayed ASD individuals as overly self‐focused, while recent studies suggest this as a struggle to navigate their social environment or distinguish themselves from others (Uddin 2011; Kanner 1968; Maenner et al. 2023).

Damasio (2010) posits the “self” as a complex structure defining social relations. It serves as the foundation of conscious awareness, with a focus on the living body. While wakefulness and mind are inherent to consciousness, the self‐imparts distinctive characteristics. Damasio's (2010) stages of self‐evolution include the autobiographical self (Kanner 1968).

“Self‐memory system” (SMS) explains the link between autobiographical memory and the self. The SMS model defines autobiographical memories as temporary mental constructs within the “working self,” a complex goal‐oriented control process. Crafting mental models aligned with objectives is a crucial function of the working self. Autobiographical memories encompass episodic memories and self‐knowledge arising from purposeful functions (Conway and Pleydell‐Pearce 2000).

While studying memory enhances our understanding of autism, it's crucial to note that ASD doesn't stem from memory difficulties. The memory model in ASD illuminates cognitive abnormalities and inner experiences. Wheeler et al. (1997) proposed that self‐consciousness relies on past personal experiences, suggesting self‐awareness is integral to episodic memory.

Studies on ASD individuals reveal atypical features in episodic memory (Powell and Jordan 1996; Crane and Goddard 2008). Some researchers suggest challenges in autobiographical episodic memory in ASD. Research indicates that while semantic autobiographical information remains intact, episodic autobiographical information diminishes (Crane and Goddard 2008; Goddard et al. 2007).

Studies report atypical development of neural structures linked to the “self” in ASD (Uddin 2011; Lyons and Fitsgerald 2013; Lombardo et al. 2013). Neural representations of “self” and “other” occur along the same pathway, involving the anterior insula, middle cingulate cortex, frontal operculum/ventral premotor cortex, and somatosensory cortex during low‐level simulative functions (Conway and Pleydell‐Pearce 2000; Bowler et al. 2011; Sato and Uono 2019a). The medial prefrontal cortex (mPFC), posterior cingulate cortex (PCC)/precuneus, and temporoparietal junction respond to both “self” and “other” states during high‐level inference‐based functions (Sato and Uono 2019a).

Current evidence shows that shared representations of these neural loops for self and other activate higher‐level cognitive functions, forming the basis for perception of the complex social world (Damasio 2010; Sato and Uono 2019a; Gülgöz et al. 2018).

The default mode network (DMN) is a broad brain network that is active during rest and particularly during mental processes such as self‐referential thinking, recalling past memories, and future‐oriented thinking. The DMN is composed of regions such as the mPFC, PCC, retrosplenial cortex (RSC), and angular gyrus, and the connections between these regions play a crucial role in forming internal mental representations (Buckner et al. 2008).

The DMN plays a significant role, especially in self‐referential information processing. Self‐referential processing involves evaluating one's thoughts, memories, and future plans in relation to oneself (Buckner et al. 2008; Northoff and Bermpohl 2004). The mPFC is key in common representations and the neural coding of self‐representation. The mPFC is associated with how an individual evaluates themselves within social contexts and makes sense of past experiences. This region becomes active during the self‐directed evaluation of personal memories.

Autobiographical memory consists of memories related to an individual's personal past, and these memories are often closely linked to self‐awareness. The DMN becomes active during such recall processes. The mPFC plays a role in evaluating personal events and interpreting the emotional context of these events (Spreng et al. 2009). The RSC, on the other hand, helps to reconstruct the spatial and temporal context of memories. Specifically, the RSC supports the recall of spatial information and context during the remembrance of personal history, allowing for a more complete representation of memories (Vann et al. 2009; Raichle and Snyder 2007).

During the recall of autobiographical memories, the integration of regions within the DMN enables the self‐directed evaluation of past events. While the mPFC imbues these memories with meaning and emotional significance, other components of the DMN, such as the RSC and PPC, organize these memories within their temporal and spatial contexts. This contributes to a more holistic recollection of memories and fosters a deeper awareness of one's personal past. The relationship between self‐referential processing and the DMN illustrates how autobiographical memory is intertwined with self‐consciousness. The DMN organizes self‐defining memories, determining how these memories shape the concept of self (Spreng et al. 2009; Rugg and Vilberg 2013). Furthermore, autobiographical memories contribute to the construction of self‐awareness through the integration of personal history and identity. By recalling and reflecting on past experiences, individuals can form a coherent narrative of their lives, which is essential for understanding themselves and their place in the world. This narrative construction not only aids in self‐identity formation but also influences emotional well‐being and social interactions, as individuals draw upon their memories to inform their current behaviors and decisions (Bluck and Alea 2008). Despite evidence of autobiographical memory impairment in ASD, there are limited neuroimaging data (Maenner et al. 2023).

Functional near‐infrared spectroscopy (fNIRS) is noninvasive neuroimaging technique measuring hemoglobin concentration change in the outer cortex using near‐infrared light (Y. Li and Yu 2016). fNIRS is reliable, portable, cost‐effective, less sensitive to head movement, and offers relatively high spatial resolution. Researchers employ fNIRS to investigate neural mechanisms in cognitive, emotional tasks, and mental states in infants and young children. Some study ASD neural abnormalities using fNIRS.

It is suggested that the development of self‐consciousness in individuals with autism is disrupted and that insufficient self‐awareness underlies the decreased autobiographical memory formation. The effect of autobiographical memory on the social development and activity of the individual is known. The main dysfunctional area in individuals with ASD is the lack of social interaction and producing appropriate behavior. This study aims to investigate how differences in self‐consciousness affect autobiographical memory in individuals with ASD and to explore the neurobiological structures underlying this relationship. Although the study utilizes a correlational design, it offers valuable insights into how deficits in self‐consciousness may relate to impairments in autobiographical memory. While causality cannot be firmly established through this design, the patterns of association that emerge can provide important clues about the underlying neurobiological mechanisms linking self‐awareness and memory. By identifying these associations, the study seeks to enhance our understanding of the intricate interplay between self‐consciousness and autobiographical memory in ASD (Table 1).

**TABLE 1:** Demographic data of the groups.

	ASD group (*n* = 4) (mean ± S.D.)	Control group (*n* = 8) (mean ± S.D.)	Test statistic (df)	Effect size	*p*
Age	19.75 ± 2.08	21.42 ± 2.37	−1.30 (10.0)	−0.79	0.320
Sex (F/M)	3/1	3/5	1.20 (10.0)	0.73	0.190
ASD scale	28.3 ± 1.25	15.9 ± 3.64	0.00	0.78	0.008*
Empathy scale	65.30 ± 0.95	75.4 ± 6.0	3.50	1.0	0.039*

Abbreviation: ASD, autism spectrum disorder; F, female; M, male; *n*, subject number; S.D., standard deviation. Bold and ‘*’ indicate that the result is statistically significant.

## 2 Materials and Methods

### 2.1 Participants

This study conducted with the approval of the Istanbul Medipol University Ethics Committee (Ethics Committee Report No: 10840098‐604.01.01‐E.14714). Participants (*n* = 12) and their guardians provided written informed consent, and there was no compensation for participation as indicated in the written informed consent.

The study comprises two groups: ASD (*n* = 4) and control group (*n* = 8). Participants are right‐handed, educated for at least 5 years, and aged 15–25. ASD group inclusion criteria are as follows: autism diagnosis, Autism Spectrum Scale score ≥ 26, and preserved language skills. The clinical profile of the autistic individuals included in the study is as follows: they experience difficulties in social interaction and exhibit repetitive behaviors but do not have any comorbid emotional diagnoses (e.g., depression). These individuals are capable of independent travel within the city, have the ability to manage shopping tasks, and receive regular therapy. In terms of language abilities, they possess intact receptive and expressive language skills, with overall language abilities well‐preserved. Control group criteria are as follows: no neurological/psychiatric diagnosis, no drug use, and Autism Spectrum Scale score < 26. ASD diagnosis by psychiatrists, approved by affiliated hospitals' Health Board and District National Education Directorate's Guidance Research Center.

The study consists of 2 stages: Stage 1 includes informing and evaluating individuals, providing LiveCam camera training, and spending 2 days with LiveCam camera recording. Phase 2 was recording of fNIRS data 36 h after camera registration was complete. After the fNIRS data were obtained, autobiographical memory tests and theory of mind tests were applied (Supporting Information S1). In the autobiographical memory test, the participants were asked to narrate five self‐defininig and five everyday memories using the instructions used (Singer and Moffitt 1991). After each memory was narrated, participants were asked to rate each memory from the Momentary Characteristic Questionnaire (Crane et al. 2009). On each of these scales, high scores were defined as self‐describing memory features, and these were used to distinguish between self‐defining and everyday memories. In addition, by applying the picture stories test to the participants, which is one of the advanced theory of mind tests, comprehension of the first‐ and second‐level belief; comprehension of first‐, second‐, and third‐level false belief; reality questions; understanding of reciprocity; understanding of deception; and understanding of cheating were evaluated. Cartoons consisting of four pictures in six sets of stories created by Brüne and Brüne‐Cohrs (2006) were shown, and the participants were asked to arrange them in the order of the event. The first goal of the study was to evaluate whether the participants with ASD could arrange the pictures according to the plot. Even if the sequence of pictures was made incorrectly by the participant, the researcher arranged the pictures in the correct order at the end of the evaluation and then asked the theory of mind questions related to the plot and made the scoring.

### 2.2 First Stage

This stage was carried out to inform all the participants (*n* = 12) to obtain and evaluate their demographic data and to provide camera training. Autism Spectrum Ratio Scale, Empathy Scale, autobiographical memory tests, and theory of mind tests were used in the evaluation (Singer and Moffitt 1991; Crane et al. 2009; Brüne and Brüne‐Cohrs 2006; Khan et al. 2015; Cutini et al. 2011).

In autobiographical memory studies, cameras are used to collect visual data and measure performance and rehabilitation (Sato and Uono 2019a). The LiveCam camera used in the research is a small digital wireless (802.11b/g/n) camera that automatically takes pictures and videos. It automatically takes a photo every 10 s. Participants continued their routine work by wearing the camera as a necklace during the time they were awake for 2 days. All participants (*n* = 12) were invited to the NIRS laboratory 36 h after the 2 days of camera recording was completed.

### 2.3 Second Stage

The images to be presented during the fNIRS registration were selected from the images obtained in Stage 1. Two types of images were presented during fNIRS recording: the “old” images were chosen from visuals that the person witnessed and recorded by the camera. “New” images were chosen from the images obtained from other participants. The task contains a total of 120 stimuli. A total of 40 old images and 80 new images were presented. In the study, the task was opted for an unbalanced design with 40 “old” images and 80 “new” images, which aligns with common practices in similar research. The primary objective of this approach is to enhance participants' focus on the target stimuli by keeping their numbers relatively low. This not only makes the targets more salient but also helps to balance the task's difficulty, thereby optimizing participants' attention and performance. Furthermore, utilizing a greater number of nontarget stimuli allows us to create a more engaging and challenging task environment without overwhelming the participants with too many target items.

**FIGURE 1:**
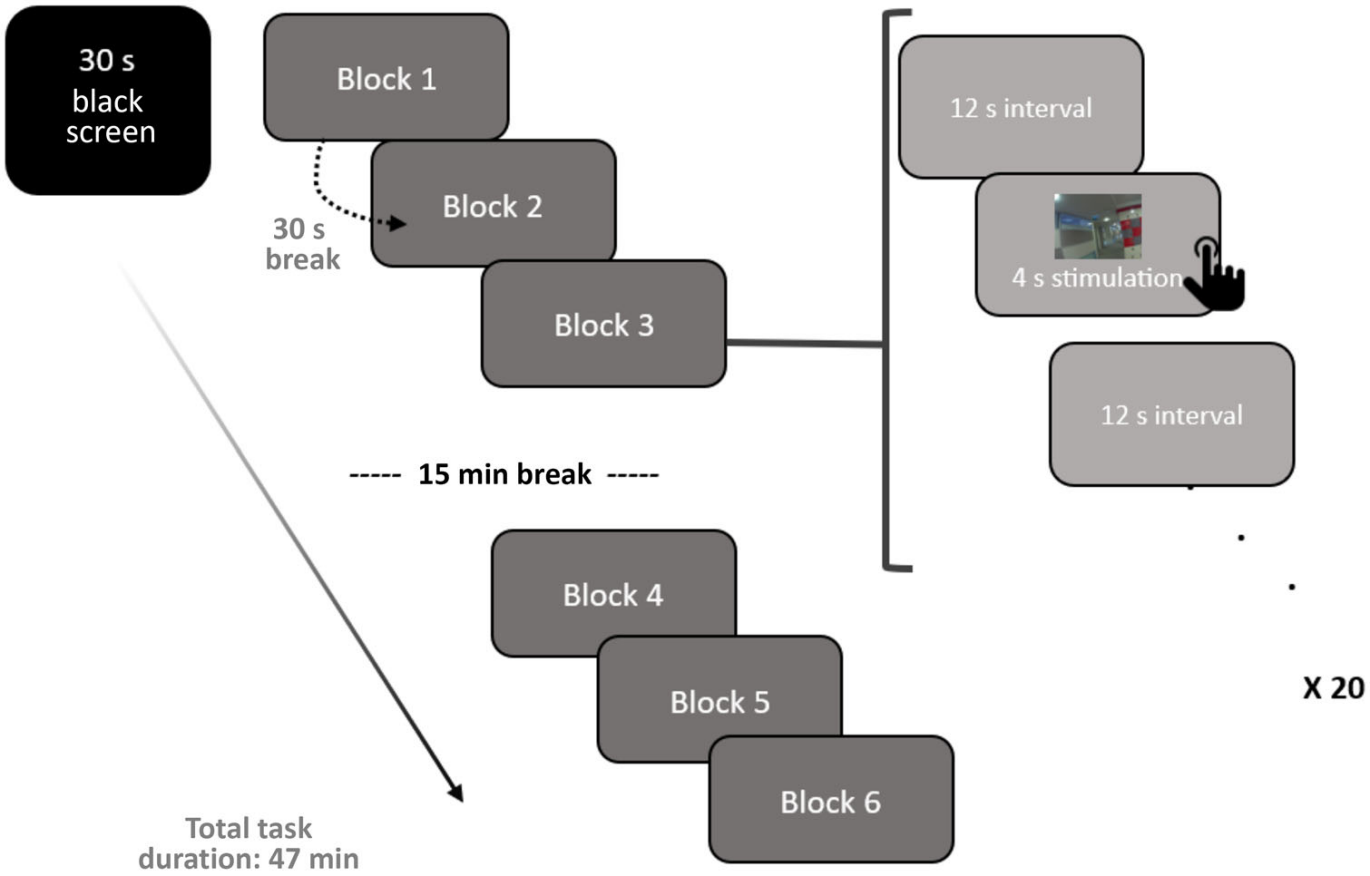
Task design.

The experimental design was created with the E‐Prime (2.0). For the task, Milton et al.’s (2011) work was modified and used. All stimuli were prepared and presented in a total of six blocks. Each block consists of 20 images, and the order of images is randomized. The visual presentation was as follows: 4 s of visual stimulus presentation and click task and 12 s of blank screen resting between stimuli. A total of 20 stimuli were shown in this way, and one block was completed, and at the end of each block, a 30‐s black screen was shown (Figure 1). Visual stimuli were displayed on a 47.5 cm × 26.8 cm monitor in full‐screen mode with a refresh rate of 60 Hz. It was placed at a viewing distance of 70 cm. The visual angle for the stimuli was measured as approximately 3.7° horizontally and 6.5° vertically. Immediately after seeing the visual stimulus, the participants were asked to press “1” button if they remembered the photos or the “z” button if they did not know. Participants did not respond in case they were not sure about the photo. Considering that individuals with autism will have difficulties, 15 min of break is given to the participants to rest after three blocks.

fNIRS recordings were performed for all ASD (*n* = 4) and control group (*n* = 8).

### 2.4 Neuroimaging and Analysis

#### 2.4.1 fNIRS Registration

**FIGURE 2:**
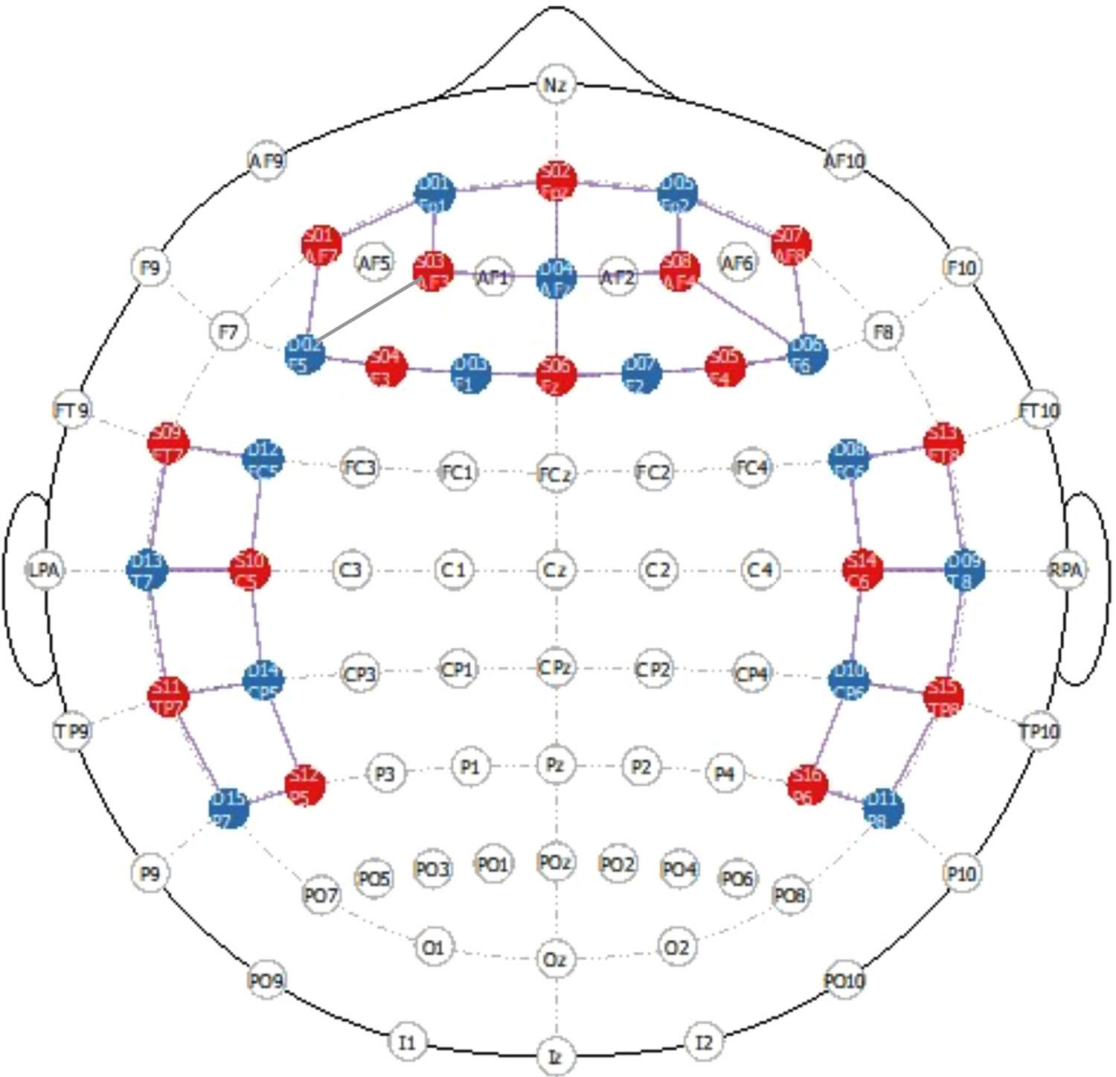
Localization of fNIRS channels (red, sources; blue, detectors; lines, channels).

fNIRS data were recorded using the fNIRS system (NIRx Medical Technologies LLC, Berlin, Germany) with the NIRStar Acquisition Software (NIRx Medizintechnik GmbH, Germany) to monitor hemodynamic activity by near‐infrared light (760 and 850 nm) during image presentation. Hemodynamic response was recorded covering the frontoparietal region using 16 sources and 15 detectors in total of 40 channels (Supporting Information S2). Positioning is prepared in accordance with the international 10–20 systems. Each source and detector are positioned 30 mm apart (Figure 2).

#### 2.4.2 fNIRS Data Analysis

fNIRS data were preprocessed using MATLAB 2019b and Homer3. The process involved converting raw data to optical density (OD). Homer3 functions addressed motion artifacts, excluding trials with such artifacts 2 s before and 15 s after stimulus presentation. A bandpass filter (0.01 Hz high pass, 0.5 Hz low pass) targeted low and high‐frequency noises. OD was converted to concentration data using the modified Beer–Lambert law, and the hmrR_GLM function calculated the hemodynamic response function (HRF) from −2.0 to 15.0 s relative to stimulus onset. Only correctly identified images were statistically analyzed for HbO data, considering the mean oxyhemoglobin value between 2 s before each stimulus and 15 s after stimulus completion.

### 2.5 Statistical Analysis

Statistical analyses were performed using IBM SPSS version 25.0. The Shapiro–Wilk test and Kolmogorov–Smirnov test were applied to evaluate the normality of the data distribution, ensuring the appropriate selection of parametric or nonparametric tests.

For nominal independent variables, the chi‐square test was employed to determine the association between categorical variables, such as evaluating the gender distribution across groups.

The independent samples *t*‐test was conducted to compare age differences between groups, as this test is suited for analyzing mean differences in continuous variables under the assumption of normal distribution.

Due to the small sample size and the scores from the Autism Spectrum Ratio Scale, Empathy Scale, theory of mind tests, and autobiographical memory tests not meeting the assumptions of normality, the Mann–Whitney *U* test was employed to compare the groups.

Given the small sample size, the Mann–Whitney *U* test was additionally employed to analyze differences across the 40 channels of fNIRS data between the two groups. Within‐group Wilcoxon signed‐rank test was used specifically to identify the differences in fNIRS data while participants viewed new and old images.

To explore the correlation between oxyhemoglobin concentration changes and performance on the autobiographical memory test, the Pearson correlation test was used.

Significance level for comparisons was set at p ≤ 0.05. For all results, significance was determined over Bonferroni correction adjusted *p* values.

## 3 Results

### 3.1 Demographic and Behavioral Findings

The age and gender distribution between the two groups was analyzed using the independent samples *t*‐test and the chi‐square test. No significant differences were found between the groups in terms of these variables.

The ASD Scale and Empathy Scale scores were compared between the two groups using the Mann–Whitney *U* test. A significant difference was observed in both scales. The autistic group had ASD scores high enough to meet the criteria for an autism diagnosis, while the control group's scores fell within the healthy range (autism group: 28.3 ± 1.25, control group: 15.9 ± 3.64, *p* = 0.008). On the Empathy Scale, the control group demonstrated significantly higher empathy abilities compared to the autistic group (autism group: 65.30 ± 0.95, control group: 75.4 ± 3.50, *p* = 0.039).

### 3.2 fNIRS Findings Obtained While Presenting the Selected Images

All channels were converted to standard Montreal Neurological Institute (MNI) coordinates. MNI coordinates were determined through the following process: first, channel locations from the fNIRS cap were obtained. These locations were then loaded into the FieldTrip neuroimaging software, where channels were aligned to an MNI template using a probabilistic estimation method. Once the MNI coordinates were extracted, anatomical labels were assigned by querying the Harvard–Oxford atlas using FSL's atlas query tool. The MNI coordinates and their corresponding anatomical labels are documented in Table S1.

#### 3.2.1 Comparison of HbO During Presentation of Selected Images Between Groups

Significant differences between the ASD and control groups for each channel were assessed using the Mann–Whitney *U* test when participants viewed the “old” images during the fNIRS recording. Similarly, the test was also used to determine whether there were any significant differences when viewing the “new” images.

When comparing the activation between groups during the presentation of “old” images that people had seen during the day, significant differences were observed in the frontopolar, orbitofrontal areas, DLPFC, Broca, pars triangularis, middle temporal gyrus (MTG), temporopolar area, Wernicke, and superior temporal gyrus (STG) regions. The mean oxyhemoglobin (HbO) concentration changes of control group were significantly higher in these regions than the ASD group.

**FIGURE 3:**
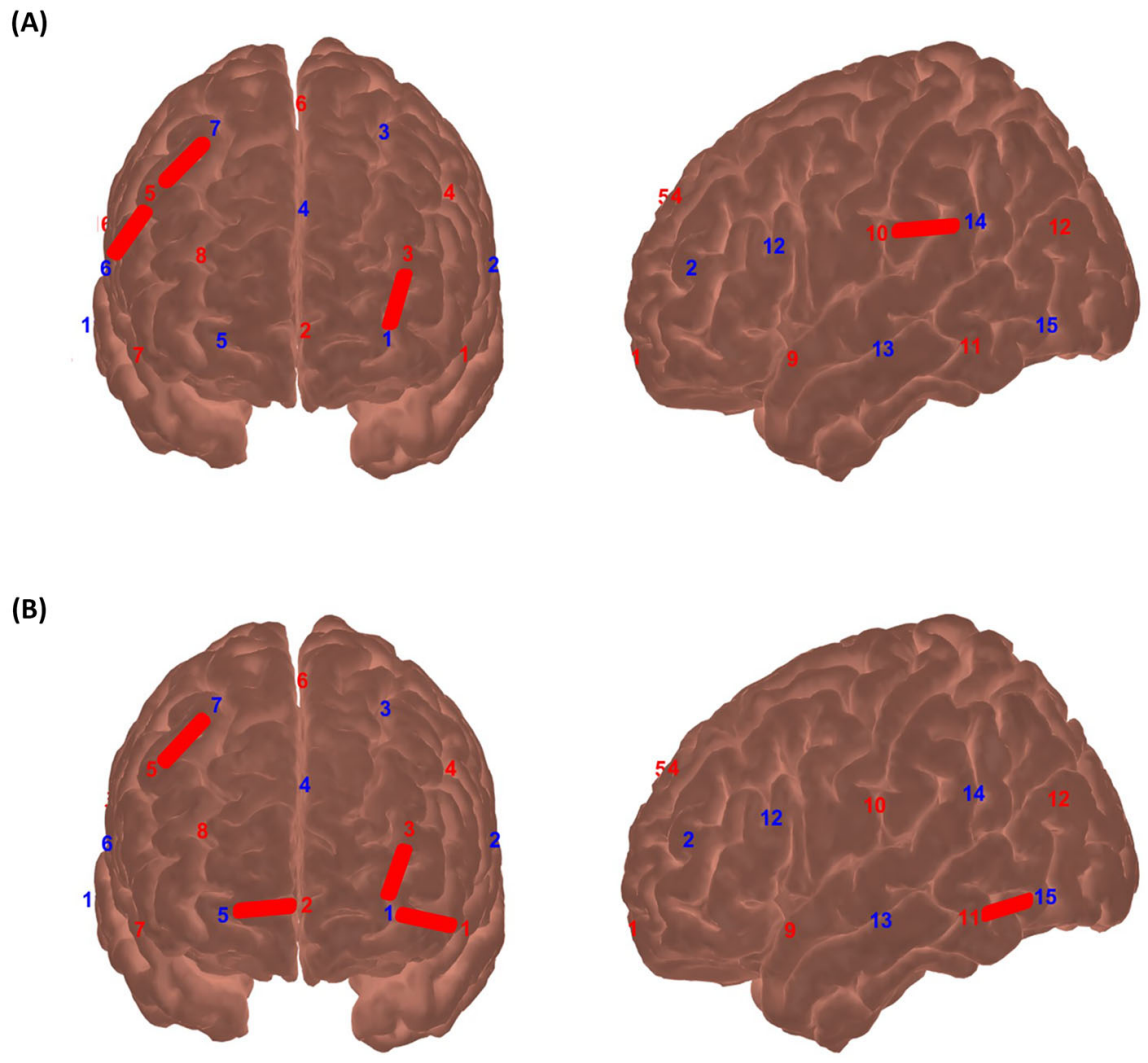
(A) Significant HbO results of the “old” image presentation between groups. (B) Significant HbO results of the “new” image presentation between groups (red, sources; blue, detectors).

When comparing the activation between groups during the presentation of “new” images that people did not see, significant differences were observed in the DLPFC/inferior prefrontal gyrus‐orbitofrontal area, frontopolar‐orbitofrontal area, DLPFC/Broca/pars triangularis‐Broca/pars triangularis, MTG/temporopolar area‐Wernicke/superior marginal gyrus/STG, and primary somatosensory cortex/subcentral area‐fusiform gyrus regions. The mean HbO of the control group was significantly higher in these regions than the ASD group (Figure 3). Statistical analysis is reported in detail in Supporting Information S3.

#### 3.2.2 Comparison of HbO During Presentation of Images Within Groups

Within each group, the Wilcoxon signed‐rank test was used to assess whether there were significant differences for each channel when viewing the “old” versus the “new” images.

When the activation of the cortex during the presentation of the “old” and the “new” images were compared in the ASD group, no significant result was seen in any brain region (*p* > 0.005).

When the activation of the cortex during the presentation of the “old” and the “new” images were compared in the control group, no significant result was seen in any brain region (*p* > 0.005).

### 3.3 Findings of Autobiographical Memory Tests

Significant differences between the ASD and control groups for the autobiographical memory test were assessed using the Mann–Whitney *U* test. The results of autobiographical memory tests are presented in Tables 2 and 3.

**TABLE 2:** Results of the autobiographical memory test items between groups.

	Self‐defining memory					Everyday memory				
	ASD group (*n* = 4) (mean ± S.D.)	Control group (*n* = 8) (mean ± S.D.)	Test statistic	Effect size	*p*	ASD group (*n* = 4) (mean ± S.D.)	Control group (*n* = 8) (mean ± S.D.)	Test statistic	Effect size	*p*
This memory reveals about me…	4.63 ± 1.75	5.80 ± 1.67	7.50	0.53	0.17	3.43 ± 1.38	4.95 ± 2.35	11.0	0.31	0.46
Vividness	5.27 ± 1.28	6.05 ± 1.43	11.0	0.31	0.44	5.27 ± 1.26	5.65 ± 2.06	12.5	0.21	0.61
Importance	4.43 ± 1.45	5.45 ± 2.04	10.5	0.34	0.38	3.23 ± 1.41	5.90 ± 1.68	2.0	0.87	0.02*
How well is the event remembered?	5.33 ± 1.12	5.85 ± 1.76	13.0	0.18	0.67	5.40 ± 1.13	5.45 ± 2.06	15.5	0.03	1.00
Thought frequency of this event	3.13 ± 1.07	5.75 ± 2.05	4.50	0.71	0.06	2.47 ± 1.22	4.90 ± 2.53	6.50	0.59	0.12
Emotionality	4.43 ± 1.63	5.50 ± 2.21	10.5	0.34	0.39	3.47 ± 1.78	5.15 ± 2.32	3.50	0.78	0.03*

Abbreviations: ASD, autism spectrum disorder; n, subject number; S.D., standard deviation. Bold and ‘*’ indicate that the result is statistically significant.

**TABLE 3:** Results of the autobiographical memory test items between groups.

	Self‐defining memory					Everyday memory				
	ASD group (*n* = 4) (mean ± S.D.)	Control group (*n* = 8) (mean ± S.D.)	Test statistic	Effect size	*p*	ASD group (*n* = 4) (mean ± S.D.)	Control group (*n* = 8) (mean ± S.D.)	Test statistic	Effect size	*p*
Length of narration	16.40 ± 2.41	68.90 ± 28.18	0.00	1.00	0.004*	11.9 ± 3.93	52.97 ± 32.35	0.00	1.00	0.04*
Specificity	0.30 ± 0.11	0.70 ± 0.25	3.00	0.81	0.03*	0.60 ± 0.28	0.82 ± 0.12	6.50	0.59	0.10
Theme	3.70 ± 0.60	3.62 ± 0.34	15.0	0.06	0.93	3.30 ± 0.80	3.28 ± 0.57	14.0	0.12	0.79
References to emotion	0.45 ± 0.19	1.02 ± 0.87	7.00	0.56	0.14	0.25 ± 0.37	0.43 ± 0.29	9.50	0.40	0.30
Sensory elements	0.05 ± 0.10	0.33 ± 0.08	1.00	0.93	0.01*	0.15 ± 0.19	0.23 ± 0.19	11.50	0.28	0.48
Self vs. other	5.15 ± 1.26	6.14 ± 0.74	6.00	0.62	0.10	5.05 ± 1.60	6.20 ± 0.38	8.00	0.50	0.19
Making meaning	0.10 ± 0.20	0.30 ± 0.35	10.50	0.34	0.36	0.0 ± 0.0	0.12 ± 0.13	6.00	0.62	0.07

**ASD**: Autism Spectrum Disorder; **n**: Subject number; **S.D**.: Standard Deviation. Bold and ‘*’ indicate that the result is statistically significant.

There were no significant differences in any of the self‐defining memory questions (*p* > 0.05). There was a significant difference only in two parts of the everyday memory questions but not in the other questions (*p* > 0.05).

When the importance of everyday memories for the individuals was evaluated, a significant difference was found between the ASD and control groups (ASD mean: 3.23 ± 1.41, control mean: 5.90 ± 1.68, *U* = 2.0, *p* = 0.02, *r* = 0.87). It was found that the control group expressed and reported the importance of the memories significantly higher than the ASD group.

When the emotional value of the everyday memories according to the subjects was evaluated, a significant difference was found between the ASD and control groups (ASD mean: 3.47 ± 1.78, control mean: 5.15 ± 2.32, *U* = 3.50, *p* = 0.03, *r* = 0.78). Significantly less emotion has been shown in the memories of the ASD group.

#### 3.3.1 Memory Length of Narration

A significant difference was found in self‐defining memory length values between the control and ASD groups (ASD mean: 16.40 ± 2.41, control mean: 68.90 ± 28.18, *U* = 0.0, *p* = 0.004, *r* = 1.00). When the everyday autobiographical memory lengths were evaluated, there were significant differences between the everyday memory length of the control group and the ASD group (ASD mean: 11.9 ± 3.93, control mean: 52.97 ± 32.35, *U* = 0.0, *p* = 0.04, *r* = 1.00). Individuals with ASD were found to have significantly shorter memories than the control group in terms of both self‐defining memory length and everyday autobiographical memory length.

#### 3.3.2 Specificity of Memories

The specificity of autobiographical memories, whether tied to a specific moment or day, was assessed for both groups. Results indicated a significant difference in memory specificity between the control group and the ASD group in the self‐defining memory category (ASD mean: 0.30 ± 0.11, control mean: 0.70 ± 0.25, *U* = 3.0, *p* = 0.03, *r* = 0.81). There was no significant difference in the specificity of everyday memories between the two groups (*p* = 0.10). The autism group exhibited significantly lower memory specificity in self‐defining memory and nonsignificantly lower memory specificity in everyday memory compared to the control group.

#### 3.3.3 Memory Theme

**FIGURE 4:**
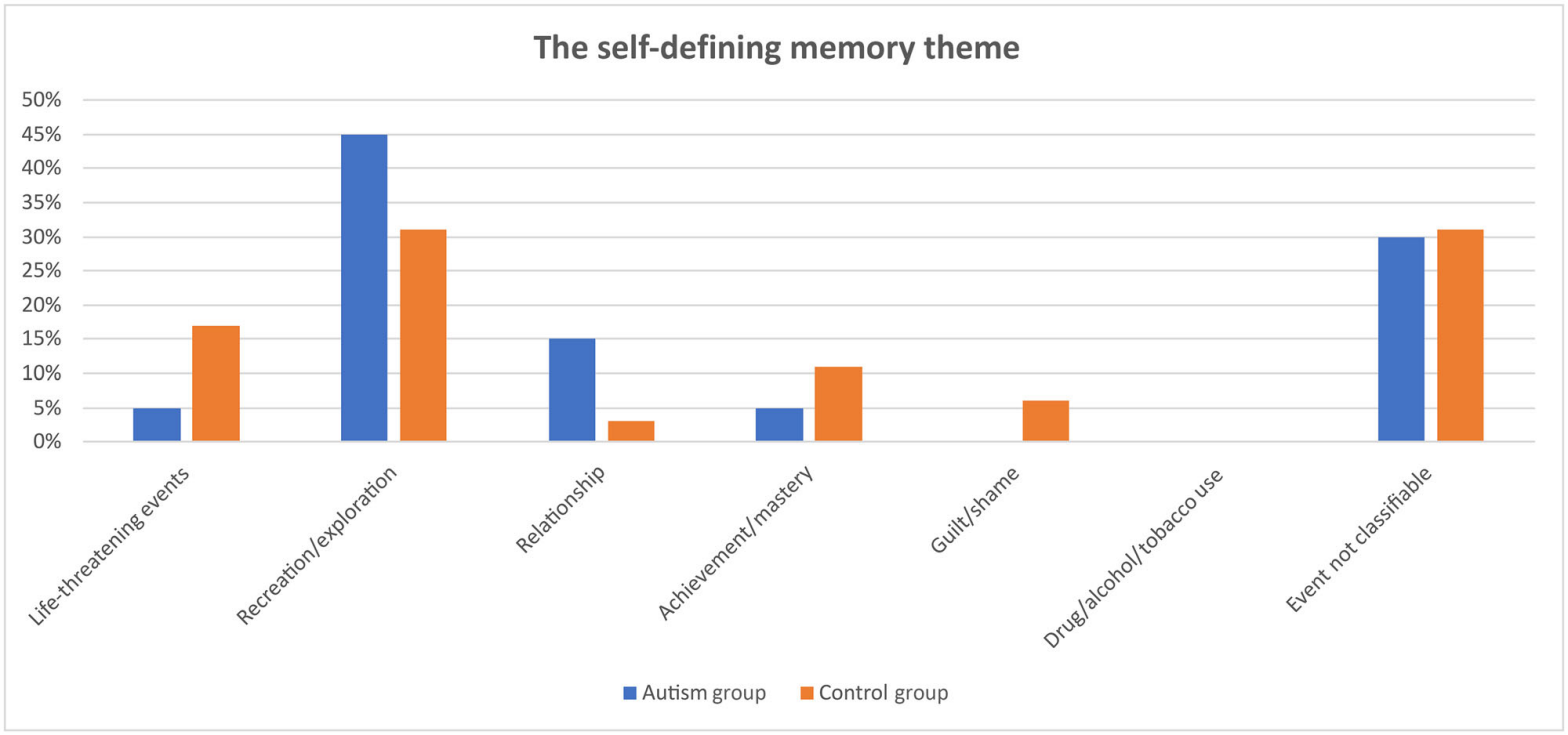
Self‐defining memory results of ASD and control groups.

In the study, subjects' narratives were categorized into seven memory types: life‐threatening events, recreation/exploration, relationship, achievement/mastery, guilt/shame, drug/alcohol or tobacco use, and events not classifiable. Life‐threatening events: these events endanger an individual's life or have the potential to cause serious harm. These types of memories are often associated with traumatic experiences and can have a significant impact on a person's life. Recreation/exploration: fun or exploratory activities. These memories include positive experiences such as traveling, exploring new places, or taking up hobbies. Relationships: memories of relationships with family, friends, or romantic partners. These types of memories reflect the importance of social bonds and emotional connections. Achievement/mastery: Memories of an individual's achievements or mastery in a particular field. These memories include achievements in areas such as education, career, or personal development. Guilt/shame: Memories of guilt or shame over past actions. These types of memories are associated with situations that conflict with the person's ethical or moral values. Drug/alcohol or tobacco use: memories of an individual's drug, alcohol, or tobacco use. These memories include experiences associated with addiction or bad habits. Events not classifiable: memories that do not fit into other categories or have no obvious theme. These types of memories may represent unique or unusual experiences that do not fit into a general classification. The two groups had no significant difference in overall memory theme scores (self‐defining memory: *p* = 0.93, everyday memory: *p* = 0.79). However, a nonsignificant difference was observed between the control group and the ASD group in certain test subsections of the self‐defining memory. The control group scored higher than the ASD group in life‐threatening events, achievement/mastery, and guilt/shame scores. The ASD group scored higher than the control group in recreation/exploration and relationship scores. (Figure 4).

#### 3.3.4 References to Emotions in Autobiographical Memories

There was no significant difference between the groups in self‐defining memories in the rate of expressing emotion and in terms of emotion content in everyday memories (*p* = 0.14, *p* = 0.30).

#### 3.3.5 Sensory Elements in Autobiographical Memories

A significant difference was found between the sensory content of the ASD group and the control group in self‐defining memories (ASD mean: 0.05 ± 0.10, control mean: 0.33 ± 0.08, *U* = 1.0, *p* = 0.01, *r* = 0.93). This indicates that individuals with ASD included significantly less sensory detail in their self‐defining memories compared to the control group. Sensory contents of everyday memories were not significantly different between the groups (*p* = 0.48).

#### 3.3.6 Self vs. Other

Results of self–other attribution were not significantly different between the ASD and the control groups in both self‐defining and everyday memory (self‐defining memory: *p* = 0.10, everyday memory: *p* = 0.19).

#### 3.3.7 Making Meaning of Memories

There was no significant difference between the groups (*p* = 0.36).

##### 3.3.7.1 Summary of Autobiographical Memory Test Results

3.3.7.1.1 Self‐Defining Memories. Self‐defining memories are deeply connected to an individual's identity, values, and life‐shaping events, often carrying emotional significance and influencing self‐perception. In the present study, no significant differences were observed between the ASD and control groups in terms of self‐defining memory questions, suggesting that both groups exhibit similarities in recalling important, identity‐related memories. However, a significant difference was found in the length of self‐defining memories, with the ASD group providing notably shorter narratives. This finding indicates that individuals with ASD offer less detail when recounting significant personal events. Furthermore, self‐defining memories in the ASD group were found to be less specific, meaning they were less likely to be tied to a particular time or event compared to the control group. No significant differences were found between the groups regarding the themes of self‐defining memories, as well as in the emotional content and expression within these memories. However, individuals with ASD included substantially fewer sensory details in their self‐defining memories compared to the control group, suggesting a reduced incorporation of sensory elements when describing important personal experiences. Additionally, there were no differences between the groups concerning attributions to self and others or the interpretation of the meaning of their memories.

3.3.7.1.2 Everyday Memory. Everyday memory encompasses the routine and ordinary events of daily life, characterized by their generally lower emotional intensity. These memories typically include daily activities, habits, and commonplace occurrences. In the present study, a significant difference was identified between the autistic and control groups in only two questions related to everyday memories, while no differences were noted in the remaining items. The ASD group reported significantly lower importance and emotional value assigned to everyday memories compared to the control group, indicating that individuals with ASD perceive daily events as less significant and less emotionally charged. Additionally, the length of everyday memories was found to be shorter in the ASD group, suggesting that individuals with ASD provide less detail when recounting daily experiences. Interestingly, no significant differences were observed between the ASD group and the control group regarding the specificity of everyday memories. This finding indicates that individuals with autism can associate their everyday memories with specific times or events to the same extent as their neurotypical counterparts. Furthermore, there were no differences in memory themes, emotional content and expression, sensory details, or attributions to self and others between the two groups. Overall, individuals with ASD exhibit shorter, less specific, less emotional, and less sensory‐rich memories. These findings indicate that autobiographical memories function differently for everyday and self‐defining memories in individuals with autism. The observed deficits, particularly in recalling significant events and incorporating sensory details, suggest substantial challenges in the construction of autobiographical memories among this population.

### 3.4 Findings of Advanced Theory of Mind Tests

Theory of mind is a social cognitive ability that allows individuals to understand the thoughts, beliefs, desires, and intentions of others. This ability is very important in terms of social interactions and interpersonal communication. Participants were asked to correctly order the parts of a six‐part cartoon story. Then, questions about the theory of mind were asked to assess their ability to understand the false beliefs of others. Significant differences between the ASD and control groups for the theory of mind test were assessed using the Mann–Whitney *U* test.

The accuracy of the sequence order across all stories was found to be significantly different between the ASD and control groups. While the control group achieved a perfect score, the ASD group scored significantly lower (ASD mean: 31.50 ± 6.75, control mean: 59.0 ± 0.0, *U* = 0.00, *p* = 0.002, *r* = 1.0).

In the first‐level order test, a significant difference was found between the ASD group and the control group (ASD mean: 2.0 ± 0.0, control group mean: 1.0 ± 0.81, *U* = 4.00, *p* = 0.010, *r* = 0.75). In the first‐level false belief test, a significant difference was found between the ASD group and the control group (ASD mean: 1.75 ± 0.95, control group mean: 3.0 ± 0.0, *U* = 4.00, *p* = 0.010, *r* = 0.75). Considering the total first‐level scores, a significant difference was found between the ASD and control groups (ASD mean: 2.75 ± 1.25, control group mean: 5.0 ± 0.0, *U* = 0.00, *p* = 0.002, *r* = 1.00).

No significant difference was found between the ASD group and the control group in the second‐level order test (ASD Mean: 1.75 ± 0.50, control group mean: 2.0 ± 0.0, *U* = 12.0, *p* = 0.21, *r* = 0.25). In the second‐level false belief test, a significant difference was found between the ASD group and the control group (ASD mean: 1.25 ± 1.25, control group mean: 3.0 ± 0.0, *U* = 4.00, *p* = 0.01, *r* = 0.75). There was a significant difference between the groups in the total second‐level score (ASD mean: 3.00 ± 1.41, control group mean: 5.0 ± 0.0, *U* = 4.00, *p* = 0.01, *r* = 0.75).

Considering the third‐level false belief test, a significant difference was found between the ASD group and the control group (ASD mean: 1.25 ± 0.95, control group mean: 3.0 ± 0.0, *U* = 0.00, *p* = 0.002, *r* = 1.00).

When the reality items were evaluated, no significant difference was found between the ASD group and the control group (ASD mean: 1.75 ± 0.50, control group mean: 2.0 ± 0.0, *U* = 12.0, *p* = 0.21, *r* = 0.25). It was found that the mental skills of the autism group showed age‐appropriate features during judgement of reality.

When the tactical deception item was evaluated, a significant difference was found between the ASD group and the control group (ASD mean: 0.50 ± 0.57, control group mean: 3.0 ± 0.0, *U* = 0.00, *p* = 0.002, *r* = 1.00).

Considering the cheating item, a significant difference was found between the ASD group and the control group (ASD mean: 0.25 ± 0.50, control group mean: 2.0 ± 0.0, *U* = 0.00, *p* = 0.002, *r* = 1.00).

In summary, we have the following:

*Sequence order accuracy*: While the control group scored perfectly on the story ordering test, the ASD group scored significantly lower. This suggests that individuals with ASD have difficulty ordering events correctly, which may affect their understanding of social situations.

*First‐level tests*: Significant differences were found between the ASD group and the control group on the first‐level ordering and false belief tests. This suggests that individuals with ASD have more difficulty understanding the beliefs of others than healthy individuals.

*Second‐level tests*: A similar pattern was observed on the second‐level tests, with the ASD group again scoring lower. However, no significant difference was found on the second‐level ordering test, suggesting that the theory of mind deficit affects false belief understanding more.

*Third‐level false belief test*: The ASD group also scored significantly lower on the third‐level false belief test, suggesting that individuals with ASD have difficulty understanding more complex social scenarios.

When assessing the elements of reality, no significant difference was found between the ASD group and the control group. This suggests that individuals with ASD have age‐appropriate reality assessment abilities.

Significant differences were also found in the tactical deception and cheating tests, suggesting that individuals with ASD have difficulty understanding or implementing social strategies such as deception in social interactions.

The theory of mind test reveals significant differences in social cognition abilities between individuals with ASD and healthy individuals. In particular, the ASD group was observed to have difficulty with sequencing events and understanding false beliefs. While they demonstrated age‐appropriate skills in reality assessment, their performance was significantly lower in tasks requiring social understanding.

**FIGURE 5:**
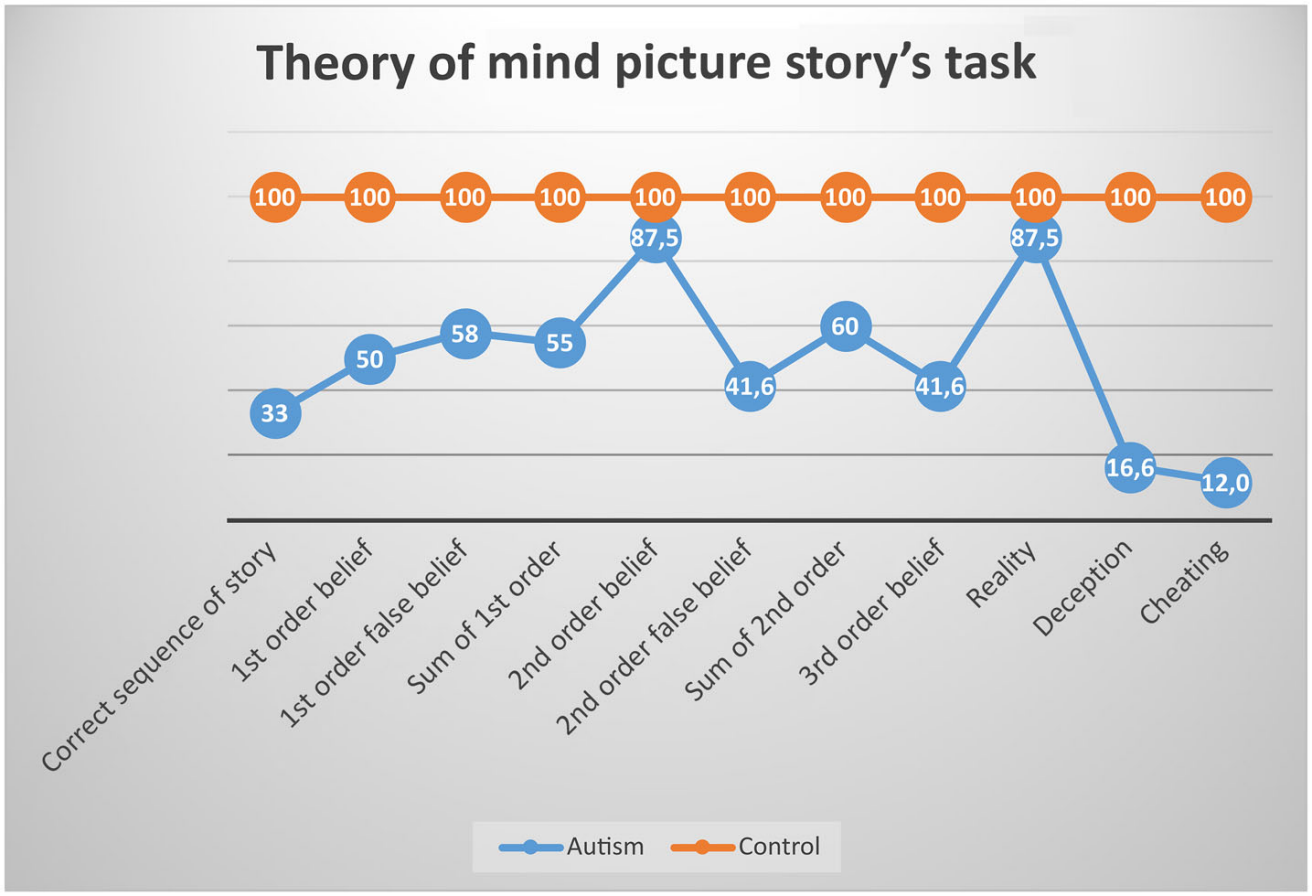
Theory of mind picture story's task results of ASD and control groups.

The findings are shown in Figure 5.

### 3.5 Oxyhemoglobin Concentration and Behavioral Data Correlation Findings

There was no significant difference between the correlation findings of the oxyhemoglobin concentration change of the ASD group and all autobiographical memory data (p < 0.05). In the control group, a significant correlation was found in many channels between the activation of both self‐defining and “other” pictures and the results of memory tests (Tables 4 and 5).

**TABLE 4:** Correlation test results between memory subtests and HbO in control group. Bold and '*' indicate that the result is statistically significant.

Channel no.	Self vs. other	Vividness	Sensory elements	Rate of recall of events	Emotionality	Theme	Momentary Characteristic Questionnaire total	References to emotion	Meaning making
Channel 2	*R* = −0.815 *p* = 0.025*	*R* = 0.8 *p* = 0.031*							
Channel 6			*R* = −0.777 *p* = 0.004*	*R* = 0.821 *p* = 0.034*	*R* = 0.786 *p* = 0.048*				
Channel 9						*R* = −0.775 *p* = 0.041*			
Channel 12		*R* = −0.946 *p* = 0.001*					*R* = −0.857 *p* = 0.024*		
Channel 15					*R* = 0.837 *p* = 0.019*				
Channel 19		*R* = −0.837 *p* = 0.019*					*R* = −0.821 *p* = 0.034*		
Channel 21	*R* = −0.778 *p* = 0.039*								
Channel 22	*R* = −0.778 *p* = 0.039*								
Channel 25							*R* = −0.821 *p* = 0.034*	*R* = −0.757 *p* = 0.049*	*R* = −0.757 *p* = 0.049*
Channel 27	*R* = −0.778 *p* = 0.039*								
Channel 31				*R* = −0.857 *p* = 0.024*					
Channel 33								*R* = −0.901 *p* = 0.006*	*R* = −0.901 *p* = 0.006*
Channel 35				*R* = −0.857 *p* = 0.024*					
Channel 37			*R* = 0.077 *p* = 0.040*						
Channel 39							*R* = −0.857 *p* = 0.024*		

**TABLE 5:** Correlation test results between memory subtests and HbO in ASD group. Bold and '*' indicate that the result is statistically significant.

Channel no.	Emotionality	How much of this memory reveals about the reporter?	Self vs. other	Sensory elements	Making meaning	References to emotion	Importance	Theme	Memory characteristic total	Specificity	How well is the event remembered?	Thought frequency	Vividness
Channel 3	*R* = −0.837 *p* = 0.019*	*R* = 0.821 *p* = 0.034*											
Channel 4			*R* = −0.77 *p* = 0.039*										
Channel 6	*R* = −0.764 *p* = 0.046*			*R* = −0.764 *p* < 0.001*	*R* = −0.77 *p* = 0.04*								
Channel 9						*R* = 0.901 *p* = 0.006*							
Channel 13							*R* = −0.775 *p* = 0.041*						
Channel 14								*R* = −0.757 *p* = 0.049*					
Channel 15							*R* = 0.775 *p* = 0.041*						
Channel 17											*R* = −0.786 *p* = 0.048*		
Channel 20						*R* = 0.811 *p* = 0.027*	*R* = 0.937 *p* = 0.002*		*R* = 0.821 *p* = 0.034*				
Channel 23				*R* = 0.823 *p* = 0.023*									
Channel 27				*R* = 0.873 *p* = 0.04*	*R* = 0.837 *p* = 0.019*								
Channel 28										*R* = −0.954 *p* < 0.001*			
Channel 29											*R* = −0.964 *p* = 0.003*	*R* = −0.883 *p* = 0.008*	
Channel 31	*R* = 0.927 *p* = 0.003*				*R* = 0.837 *p* = 0.019*								
Channel 33						*R* = −0.955 *p* < 0.001*			*R* = −0.893 *p* = 0.012*		*R* = −0.857 *p* = 0.024*		
Channel 35	*R* = 0.818 *p* = 0.024*	*R* = 0.821 *p* = 0.034*		*R* = 0.767 *p* = 0.044*									*R* = −0.847 *p* = 0.010*
Channel 37	*R* = 0.764 *p* = 0.046*			*R* = 0.767 *p* = 0.044*									
Channel 38								*R* = 0.847 *p* = 0.016*		*R* = −0.767 *p* = 0.044*			
Channel 39						*R* = −0.847 *p* = 0.016*							
Channel 40							*R* = 0.775 *p* = 0.041*						

## 4 Discussion

This study explores the connection between self‐development, neurobiological structures, and autobiographical memory in individuals with autism. Autistic subjects underwent assessments using the theory of mind test and autobiographical memory task. Additionally, neural structures linked to recalling autobiographical memories were investigated using LiveCam and fNIRS, a first‐time approach for autistic subjects.

Based on the study's findings, our hypothesis that differences in self‐consciousness are associated with impairments in autobiographical memory in individuals with ASD is supported to a significant extent. The results revealed that individuals with ASD had shorter, less specific, and less sensory‐rich autobiographical memories compared to the control group. This aligns with the idea that deficits in self‐consciousness may impact memory construction. The significant differences in oxyhemoglobin (HbO) concentration levels between the ASD and control groups, particularly in regions associated with self‐awareness and memory processing (e.g., DLPFC, Broca's area, and temporal regions), suggest that neurobiological mechanisms indeed underlie this relationship. Although our study used a correlational design and causality cannot be firmly established, the observed associations between reduced self‐consciousness and autobiographical memory impairments, as well as the distinct neural activation patterns, provide valuable insights into the underlying neurobiological structures linking self‐awareness and memory in ASD.

Reduced theory of mind responses, except for reality and reciprocity aspects, in individuals with autism indicate difficulties in understanding others' beliefs, feelings, and wishes, contributing to challenges in communication and social interaction. Chiu et al. (2023) suggested that autism involves struggles with monitoring intentions in social interactions, resulting in impaired theory of mind, introspection, and self‐referential processing. Similarly, the findings in the theory of mind test in this study reveal that individuals with autism have problems in ordering events and understanding false beliefs, and although individuals with autism show similar skills to healthy individuals in reality assessment, their performance in social understanding tasks is significantly lower.

Autobiographical memory, shaped by self‐perception, plays a crucial role in how individuals recall and interpret life events. It significantly influences identity and self‐understanding, as well as the ability to relate to others and navigate social environments. In the current study, the performance of individuals with ASD on autobiographical memory tasks offered valuable insights into how self‐consciousness and memory construction are intertwined in this population. Self‐defining memories, which reflect an individual's identity, values, and life‐shaping experiences, typically hold emotional significance and contribute to self‐perception. The findings from our study revealed no significant differences between the ASD and control groups in recalling self‐defining memories. Both groups demonstrated a similar ability to recall memories associated with personal identity. However, there was a marked difference in the length of the narratives, with the ASD group providing notably shorter accounts, which suggests that individuals with ASD offer fewer details when describing significant personal events. Additionally, their self‐defining memories were found to be less specific, meaning they were less likely to be tied to a particular time or event compared to the control group. While no significant differences were observed regarding the themes, emotional content, or expression of these memories, individuals with ASD included substantially fewer sensory details. This aligns with existing research, such as that of Millward et al. (2000), which indicates that individuals with ASD often recall fewer real‐life episodes due to a limited sense of “self to experience” (Wheeler and Stuss 2003; Pfeifer and Peake 2012). The absence of certain memory themes, such as success, guilt/shame, and threat‐related memories, further supports the notion that autobiographical memory in individuals with ASD may lack the affective and motivational content seen in typical development. Wheeler et al. (1997) highlight the role of autonoetic consciousness in recalling personal events, and in individuals with ASD, this self‐awareness is thought to be diminished (Lukito et al. 2020). This may explain why individuals with ASD struggle to construct vivid and emotionally rich self‐defining memories. Everyday memory, which encompasses routine, ordinary events with generally less emotional intensity, also revealed important differences between the ASD and control groups. While most of the everyday memory items showed no significant differences between the two groups, individuals with ASD assigned lower importance and emotional value to daily memories, indicating that they perceive such events as less significant and less emotionally engaging. Similar to self‐defining memories, their everyday memory narratives were shorter and less detailed, though they exhibited a similar capacity to associate these memories with specific times or events compared to their neurotypical peers. Interestingly, there were no differences in terms of memory themes, emotional content, sensory details, or attributions to self and others between the ASD and control groups in everyday memories. Despite this, the observed pattern of shorter, less specific, and less sensory‐rich memories in individuals with ASD aligns with broader findings in the literature, suggesting that autobiographical memory formation is uniquely impacted in this population. This is consistent with developmental theories that link the emergence of autobiographical memory to self‐awareness, language development, narrative abilities, and theory of mind. In ASD, however, the experience of “the self” seems to lack the affective and motivational richness necessary for robust memory construction (Wheeler et al. 1997; Lukito et al. 2020; X. Li et al. 2021). The findings from this study highlight that individuals with ASD demonstrate significant challenges in constructing both self‐defining and everyday memories, particularly in terms of narrative length, specificity, and sensory richness. These results are consistent with theories that suggest a disruption in self‐consciousness in individuals with ASD, leading to difficulties in autobiographical memory formation.

In the study, brain areas showing a notable difference in HbO levels during the presentation of “old” images in the autistic group coincide with regions involved in advanced cognitive processes, including the frontopolar and orbitofrontal areas. Autobiographical memory involves personal decision‐making, planning, and social awareness, contributing to holistic self‐expression and continuity over time (Zhu et al. 2022). Theory of mind, a complex cognitive process allowing the understanding of mental states in oneself and others, is vital for social interaction and communication. Executive functioning difficulties in children with autism may hinder the initiation, planning, and organization of autobiographical memory retrieval, impacting coherent narrative formation about past experiences. The frontopolar area, a central hub for integrating information across the brain, plays a vital role in forming a cohesive autobiographical memory. Dysfunction in this area may contribute to challenges in memory integration in children with ASD. Studies note altered activity or connectivity in the orbitofrontal region in children with autism, potentially impacting their processing of social and emotional information (Petrican et al. 2020). Difficulties in understanding social cues hinder individuals with autism from developing a consistent self in relationships. Struggles with perspective‐taking and recognizing emotions in themselves and others impede the development of a strong self‐relationship. Orbitofrontal region dysfunction can impact self‐awareness and introspection, as it evaluates internal states and self‐knowledge (Petrican et al. 2020; Rolls et al. 2022). In the study, low activation in channels representing the frontopolar‐orbitofrontal area in the autism group aligns with significant differences in autobiographical memory results, including self‐definition, memory specificity, recall quality, memory importance, narration length, and theory of mind test outcomes.

In the autistic group, another notable difference in activation occurs in the DLPFC/Broca/pars triangularis–DLPFC and DLPFC/Broca/pars triangularis–Broca/pars triangularis channels. The DLPFC, crucial for autobiographical memory retrieval and organization, aids in forming and integrating memories into narratives (Sato and Uono 2019b). Executive function difficulties in children with autism, potentially linked to atypical DLPFC development, may impede initiating and constructing autobiographical memory recall. This can manifest as challenges in remembering specific events, arranging memories chronologically, and expressing feelings about past experiences, resulting in fragmented self‐narratives. Although Broca isn't directly tied to autobiographical memory, it plays a vital role in linguistically expressing memories. Atypical development or functioning of Broca in individuals with autism may hinder verbalizing autobiographical memories and self‐experiences, obstructing the formation of a coherent self‐narrative (Shibata et al. 2021). The pars triangularis is crucial for structuring language and is vital in constructing coherent autobiographical narratives by contributing to grammatical and syntactic aspects (Shibata et al. 2021). In children with autism, issues in its functioning can disrupt language syntax, impacting the organization and expression of autobiographical memories and potentially affecting narrative coherence and self‐identity conveyance. Overall, the DLPFC, Broca, and pars triangularis are pivotal in language processing and executive functions and crucial for autobiographical memory processes. Atypical development in these regions may hinder self‐perception and autobiographical memory narration in autistic children, leading to difficulties in remembering, organizing, and expressing personal experiences. From a theory of mind perspective, the DLPFC supports understanding others' mental states, aiding in tracking social information, and considering different viewpoints. Language, facilitated by Broca and pars triangularis, is vital for expressing and understanding mental states. The significant HbO activation in the autistic group in the study aligns with autobiographical memory and theory of mind test findings.

In the ASD group, the channel reflecting the MTG/temporopolar area–Wernicke/superior marginal gyrus/STG exhibited significant HbO change to old photographs. The MTG is a complex brain region involved in language comprehension, semantic memory, and audiovisual integration (Shibata et al. 2021). Crucial for accessing and processing information about word meanings, objects, and concepts, MTG is responsible for retrieving semantic information related to personal experiences in autobiographical memory (Petrican et al. 2020). Atypical functioning in MTG in individuals with autism may hinder access and integration of semantic information from autobiographical memories, resulting in shallow and less rich autobiographical narration. This can impact their sense of self and the ability to construct a coherent self‐identity.

Wernicke is critical for language comprehension and semantic processing (Shibata et al. 2021). Atypical development or functioning in individuals with autism may lead to difficulties understanding complex oral and written language, limiting their grasp of the meaning and significance of experiences, and hindering coherent self‐formation. The superior marginal gyrus, linked to self‐awareness and perspective, contributes to self‐understanding and knowledge (Petrican et al. 2020). Changes in this area can impact self‐awareness and integrate self‐related information into autobiographical memories, affecting coherent self‐expression and potentially self‐concept and identity. The STG is involved in auditory processing functions. Differences in the STG in individuals with autism may lead to difficulties in processing auditory information, affecting understanding and recall of autobiographical memories during verbal expressions (Cechmanek et al. 2018; van Kooten et al. 2008). Significant activation differences in these areas align with autobiographical memory and theory of mind test results.

The study compared fNIRS activation results for “new” photos unfamiliar to the ASD and control groups. Significant HbO in the ASD group was observed in the DLPFC/Inferior prefrontal gyrus and the orbitofrontal area. Additionally, another noteworthy channel on the primary somatosensory cortex/subcentral area–fusiform gyrus was identified. The inferior prefrontal gyrus, linked to language processing and semantic memory, plays a role in semantic integration, understanding, and recall of words and concepts (Shibata et al. 2021). Its atypical functioning may impact how individuals with ASD retrieve and express personal memories, and it is also involved in social cognition. Activation differences in this region may affect the self‐narrations and social interactions of individuals with autism.

The primary somatosensory cortex, though not directly tied to autobiographical memory, plays a role in sensory experiences linked to personal memories, like tactile sensations. Processing somatosensory information, while not a primary challenge in ASD, may impact how autistic individuals experience and recall autobiographical memories (Cechmanek et al. 2018). The subcentral area integrates sensory information, contributing to overall sensory processing and body awareness in autobiographical experiences. Atypical sensory processing in ASD's subcentral region can affect how they perceive and integrate sensory information during personal experiences, influencing the sensory aspects of autobiographical memories. The fusiform gyrus, crucial for face perception and recognition, plays a vital role in social cognition (van Kooten et al. 2008; Apicella et al. 2013; Baron‐Cohen et al. 2001). In autobiographical memory and self, it may be involved in visual processing of self‐related stimuli and recognizing others' faces in personal experiences, affecting how individuals with autism remember and interpret social interactions in their narratives. Differences in face processing and atypical fusiform gyrus function in ASD contribute to difficulties in face recognition and emotion interpretation. Collectively, these regions are implicated in sensory processing, somatosensory integration, and visual recognition related to autobiographical memory, self‐experiences, and social cognition in children with autism. The study data supports the idea that sensory experiences, body awareness, and social perception contribute to autobiographical memories and self‐concept in autism. Addressing sensory processing difficulties and promoting social cognition is crucial for improving autobiographical memory and self‐experiences in autistic children (Baron‐Cohen et al. 2001). The sensory content of autobiographical memories plays a crucial role in how individuals encode and recall personal experiences. In the current study, individuals with ASD were found to provide significantly fewer sensory details in the self‐defining compared to the control group. This aligns with research suggesting that sensory processing difficulties are a central feature of ASD, impacting how memories are constructed and recalled. Millward et al.’s (2000) findings, which indicate that individuals with ASD often recall fewer real‐life episodes, may be linked to these sensory processing challenges. Recent studies emphasize that sensory experiences, body awareness, and social perception are critical for the development of autobiographical memory and self‐concept in individuals with ASD. Specifically, regions involved in sensory processing, somatosensory integration, and visual recognition—such as the PCC and precuneus—are implicated in how sensory input is integrated into autobiographical memory. The diminished sensory richness observed in the ASD group suggests a possible neurobiological disruption in these regions, which may hinder the ability to form detailed, vivid memories tied to sensory experiences.

Examining the statistical correlation between memory and fNIRS results, no significant difference was found in the ASD group. In the control group, a significant correlation was observed in many channels between the activation of self‐defining and “other” pictures and memory test results. These correlated channels overlapped with the previously discussed brain regions, supporting the impact of these areas.

In summary, atypical functioning or connectivity in these brain regions contributes to difficulties in autobiographical memory recall, language comprehension, self‐awareness, and self‐relationship in individuals with ASD. Struggles in accessing, integrating, and expressing autobiographical memories impact forming a coherent self‐narrative and stable self‐identity. Interventions targeting language comprehension, semantic memory, self‐awareness, and auditory processing can aid children with autism in developing more integrated autobiographical memory narratives and self‐concepts. Personalized approaches, considering individual strengths and challenges, are crucial for effective support. The study highlights the link between self‐consciousness and autobiographical memory, revealing the neurobiological structures influencing memory and self‐consciousness in autistic individuals. Developmental factors can significantly influence both memory and self‐referential processing, as cognitive and social abilities typically evolve. The variation in age may lead to differences in the maturity of these processes, potentially confounding our results. These results further reinforce the intricate interplay between cognitive and neural processes in shaping autobiographical memory in individuals with ASD. The main limitations of the study are sample size and age range limit. Future research should aim to expand the participant pool to include a broader age range and a larger number of individuals to better capture the developmental nuances associated with self‐consciousness and autobiographical memory in ASD. Additionally, it should focus on conducting further analyses that investigate interaction effects between “old” and “new” items, as this may provide deeper insights into the neural mechanisms underlying memory processes in both ASD and control groups.

## Author Contributions

**Yesim Unveren**: conceptualization, investigation, writing – original draft, writing – review and editing, methodology. **Mevhibe Saricaoglu**: writing – original draft, methodology, visualization, writing – review and editing, formal analysis, data curation. **Ece Zeynep Karakulak**: writing – original draft, visualization, writing – review and editing, methodology, data curation. **Lütfü Hanoğlu**: conceptualization, investigation, writing – original draft, methodology, writing – review and editing, supervision.

## Ethics Statement

This study was conducted with the approval of the Istanbul Medipol University Ethics Committee (Ethics Committee Report No: 10840098‐604.01.01‐E.14714).

## Consent

Participants and their guardians provided written informed consent, and there was no compensation for participation as indicated in the written informed consent.

## Conflicts of Interest

The authors declare no conflicts of interest.

### Peer Review

The peer review history for this article is available at https://publons.com/publon/10.1002/brb3.70349

## Supporting information

Supporting Information

## Data Availability

The datasets used and analyzed during the current study are available from the corresponding author upon reasonable request.
