# Supplementary material for: Investigation of the Relationship Between Self‐Consciousness and Autobiographic Memory in Individuals with Autism Spectrum Disorders with LiveCam and fNIRS
Source: Brain Behav. 2025 Feb 28;15(3):e70349. doi: 10.1002/brb3.70349 (PMC11870838; doi:10.1002/brb3.70349)
Supplement: Supplementary file 1 — Supporting Information [file BRB3-15-e70349-s001.docx]

**Supplementary Material 1**

**Autobiographical Memory Task**

**Memory Characteristics Questionnaire**

(a) This memory reveals or says about me... (1=not very much, 7=a lot)

(b) I remember this event… (1=hardly to, 7=very well)

(c) On a scale of vividness, this memory is... (1=not at all, 7=extremely)

(d) On a scale of importance, this memory is.. (1=not at all, 7= extremely)

(e) Since it has all happened, I have thought about the event… (1= not at all, 7= many times) (f) On a scale of emotionality, this memory is... (1= not at all, 7= extremely)

The number of memories expressed in each category and the length of the narratives were also evaluated. Narrative features were also coded by the researcher according to the following features:

**Specificity**

A reference to a specific autobiographical memory, that is, an individual event lasting no longer than a day in the narrative (e.g. my first day at university). Categorical memories; (e.g., Monday morning lessons) repeated without reference to a single event received a score of 0.

**Theme**

Memories were placed in one of the following 7 memory theme categories (17).

1. Life-threatening events: This category consists of narratives that include topics describing life and death or physical and mental well-being. For example, deaths, accidents, injuries or illnesses, sexual or physical assaults, and severe mental or physical illness.

2. Recreation/Exploration: These memories include those that focus on recreation, exploration, and play. For example, hobbies, parties, outings, travels, vacations or sports activities.

3. Relationship: Relationship narratives involve a specific interpersonal relationship (usually involving a parent or friend). It should mention a relationship or an event containing cathexis for the other person. For example, first love, ending the relationship, separation, reconciliation, intimacy.

4. Achievement/mastery: Narratives should highlight attempts at mastery or success on a topic, regardless of outcome. For example, passing or failing a test, learning to drive, believing in a new religion, starting a new life (such as leaving family).

5. Guilt/shame: The narratives in this category highlight the issues of right and wrong. For example, feelings of remorse for lying to someone or for hurting someone, or events that have been morally or ethically decided.

6. Substance, alcohol or tobacco use: These narratives are those that emphasize the use of drugs, alcohol and tobacco for the purpose of seeking entertainment or excitement. For example, the first cigarette or substance use, the experience of being very drunk.

7. Unclassifiable events: This category includes other narratives that do not fit into the above categories.

**References to Emotion**

Since autism is associated with disturbances in emotional processing, each reference to an emotion in narratives with emotional material received 1 point.

**Sensory Elements**

Unusual sensory processing has been commonly reported in individuals with autism. Each sensory item found in the narratives received a score of 1 (e.g. I still remember the scent of flowers).

**Self vs Other**

Previous research has shown that individuals with autism generally cannot use information about the self to facilitate access to memory (18). Therefore, whether the memory is focused on “self” or the “other” is scored on a seven-point Likert scale (1= the memory is mostly focused on others, 7= the memory is mostly focused on self).

**Meaning Making**

Two types of inference were coded: learning and gaining insight. Inference indicates that they learned a particular lesson from the event that might have implications for their behavior in other similar situations in the future (e.g. My uncle died of alcoholism. I learned not to be addicted to any substance that would control me). Gaining insight involves understanding a situation that goes beyond the specific behavior described and concerns a wider area of one's life (e.g. my brother passed away last year. I've learned to live life to the fullest and be thankful for every day). Each moment involving these features received a score.

**Supplementary Material 2**

**Table S1.** Brodmann areas of fNIRS channels

| **Channel No.** | **Soruce-Detector Optodes** | **Location** | **Brodmann Area (Source-Detector)** |
| --- | --- | --- | --- |
| Channel 1 | S1-D1 | AF7-FP1 | DLPFC/Inferior Prerontal Gyrus- Orbitofrontal Area |
| Channel 2 | S1-D2 | AF7-F5 | DLPFC/Inferior Prerontal Gyrus-Broca/Pars Triangularis |
| Channel 3 | S2-D1 | FPz-FP1 | Frontopolar Area/Orbitofrontal Area-Orbitofrontal Area |
| Channel 4 | S2-D4 | FPz-AFz | Frontopolar Area-Frontopolar Area |
| Channel 5 | S2-D5 | FPz-FP2 | Frontopolar Area-Orbitofrontal Area |
| Channel 6 | S3-D1 | AF3- FP1 | Frontopolar Area-Orbitofrontal Area |
| Channel 7 | S3-D2 | AF3-F5 | Frontopolar Area-Broca/Pars Triangularis |
| Channel 8 | S3-D4 | AF3-AFz | Broca/Pars Triangularis-Frontopolar Area |
| Channel 9 | S4-D2 | F3-F5 | DLPFC/Broca/Pars Triangularis-Broca/Pars Triangularis |
| Channel 10 | S4-D3 | F3-F1 | DLPFC/Broca/Pars Triangularis-DLPFC |
| Channel 11 | S5-D6 | F4-F2 | DLPFC/Broca/Pars Triangularis-DLPFC |
| Channel 12 | S5-D7 | F4-F6 | DLPFC/Broca/Pars Triangularis-Broca/Pars Triangularis |
| Channel 13 | S6-D3 | Fz-F1 | DLPFC/Frontal Eye Field-DLPFC |
| Channel 14 | S6-D4 | Fz-AFz | DLPFC/Frontal Eye Field-Frontopolar Area |
| Channel 15 | S6-D7 | Fz-F6 | DLPFC/Frontal Eye Field-Broca/Pars Triangularis |
| Channel 16 | S7-D5 | AF4-FP2 | Frontopolar Area-Orbitofrontal Area |
| Channel 17 | S7-D6 | AF4-F2 | Frontopolar Area-DLPFC |
| Channel 18 | S8-D4 | AF8-AFz | DLPFC/Inferior Prefrontal Gyrus-Frontopolar Area |
| Channel 19 | S8-D5 | AF8-FP2 | DLPFC/Inferior Prefrontal Gyrus-Orbitofrontal Area |
| Channel 20 | S8-D6 | AF8-F2 | DLPFC/Inferior Prefrontal Gyrus-DLPFC |
| Channel 21 | S9-D12 | FT7-FC5 | Middle Temporal Gyrus/Temporopolar Area-Broca/Pars Opercularis/Premotor/Supplementary Area |
| Channel 22 | S9-D13 | FT7-T7 | Middle Temporal Gyrus/Temporopolar Area-Middle Temporal Gyrus |
| Channel 23 | S10-D12 | FT8-FC5 | Middle Temporal Gyrus/Temporopolar Area-Broca/Pars Opercularis/Premotor/Supplementary Area |
| Channel 24 | S10-D13 | FT8-T7 | Middle Temporal Gyrus/Temporopolar Area-Middle Temporal Gyrus |
| Channel 25 | S10-D14 | FT8-CP5 | Middle Temporal Gyrus/Temporopolar Area- Wernicke/Superior Marginal Gyrus/Superior Temporal Gyrus |
| Channel 26 | S11-D13 | C5-T7 | Primer Somatosensory Cortex/Subcentral Area-Middle Temporal Gyrus |
| Channel 27 | S11-D14 | C5-CP5 | Primer Somatosensory Cortex/Subcentral Area-Wernicke/Superior Marginal Gyrus/Superior Temporal Gyrus |
| Channel 28 | S11-D15 | C5-P7 | Primer Somatosensory Cortex/Subcentral Area-Fusiform Gyrus |
| Channel 29 | S12-D14 | C6-CP5 | Primer Somatosensory Cortex/Subcentral Area/Superior Temporal Gyrus-Wernicke/Supramarginal Gyrus/Superior Temporal Gyrus |
| Channel 30 | S12-D15 | C6-P7 | Primer Somatosensory Cortex/Subcentral Area/Superior Temporal Gyrus-Fusiform Gyrus |
| Channel 31 | S13-D8 | TP7-FC6 | Inferior Temporal Gyrus/Middle Temporal Gyrus/Fusiform Gyrus-Premotor/Supplementary Motor Area/ Broca/Pars Opercularis |
| Channel 32 | S13-D9 | TP7-T8 | Inferior Temporal Gyrus/Middle Temporal Gyrus/Fusiform Gyrus-Middle Temporal Gyrus |
| Channel 33 | S14-D8 | TP8-FC6 | Inferior Temporal Gyrus/Middle Temporal Gyrus/Fusiform Gyrus-Premotor/Supplementary Motor Area/ Broca/Pars Opercularis |
| Channel 34 | S14-D9 | TP8-T8 | Inferior Temporal Gyrus/Middle Temporal Gyrus/Fusiform Gyrus-Middle Temporal Gyrus |
| Channel 35 | S14-D10 | TP8-CP6 | Inferior Temporal Gyrus/Middle Temporal Gyrus/Fusiform Gyrus-Wernicke/Supramarginal Gyrus/Superior Temporal Gyrus |
| Channel 36 | S15-D9 | P5-T8 | Wernicke/Angular Gyrus-Middle Temporal Gyrus |
| Channel 37 | S15-D10 | P5-CP6 | Wernicke/Angular Gyrus-Wernicke/Supramarginal Gyrus/Superior Temporal Gyrus |
| Channel 38 | S15-D11 | P5-P8 | Wernicke/Angular Gyrus-Fusiform Gyrus |
| Channel 39 | S16-D10 | P6-CP6 | Wernicke/Angular Gyrus-Wernicke/Supramarginal Gyrus/Superior Temporal Gyrus |
| Channel 40 | S16-D11 | P6-P8 | Wernicke/Angular Gyrus-Fusiform Gyrus |

**Suplementary Material 3**

- - 1. **Comparison of HbO During Presentation of Selected Images Between Groups**

When comparing the activation between groups during the presentation of “old” images that people had seen during the day, significant differences were observed in the frontopolar and orbitofrontal areas, DLPFC, Broca’s area, Pars Triangularis, Middle Temporal Gyrus, Temporopolar area, Wernicke’s area and Superior Temporal Gyrus regions (Channel 6: t (10.0) =1.87 p= .031 OG Mean: -1.04±0.67, HG Mean ± S.D.:1.13±2.25; Channel 11: t (10.0) =3.53 p= .001 OG Mean:-1.68±0.46, HG Mean ± S.D.:0.75±1.31; Channel 12: t (10.0) =2.56 p= .028 OG Mean:-1.71±3.07, HG Mean ± S.D.:2.01±2.00; Channel 25: t (10.0) =3.26 p= .009 OG Mean: -1.68±2.81, HG Mean ± S.D.:2.16±1.38). The mean oxyhemoglobin concentration changes (HbO) of the healthy group were significantly higher in these regions than the ASD group. (Table 4, Table 5)

When comparing the activation between groups during the presentation of “new” images that people did not see, significant differences were observed in the DLPFC/Inferior Prefrontal Gyrus-Orbitofrontal Area, Frontopolar Area-Orbitofrontal Area, DLPFC/Broca/Pars Triangularis-Broca/Pars Triangularis, Middle Temporal Gyrus/Temporopolar Area-Wernicke/Superior Marginalis Gyrus/Superior Temporal Gyrus, Primary Somatosensory Cortex/Subcentral Area-Fusiform Gyrus regions (Channel 1: t (0.0) =3.10 p= .006 OG Mean:-2.43±1.16, HG Mean ± S.D.:0.72±1.83; Channel 5: t (10.0) =2.57 p= .031 OG Mean:-2.99±1.46, HG Mean ± S.D.:-0.45±1.67; Channel 6: t (10.0) =2.64 p= .024 OG Mean:-2.59±1.81, HG Mean ± S.D.:0.80±2.20; Channel 12: t (10.0) =3.13 p= .011 OG Mean:-2.50±2.68, HG Mean ± S.D.:1.38±1.66; Channel 25: t (10.0) =5.73 p= .001 OG Mean:-2.99±1.41, HG Mean ± S.D.:2.12±1.47; Channel 28: t (10.0) =2.38 p= .039 OG Mean:-2.85±2.03, HG Mean ± S.D.:-0.11±1.80) (Table 4,5). The mean HbO of the healthy group were significantly higher in these regions than the autistic group (Figure 1).
